# Association of *TP53 *codon 72 polymorphism and the outcome of adjuvant therapy in breast cancer patients

**DOI:** 10.1186/bcr1682

**Published:** 2007-05-30

**Authors:** Tatsuya Toyama, Zhenhuan Zhang, Mariko Nishio, Maho Hamaguchi, Naoto Kondo, Hirotaka Iwase, Hiroji Iwata, Satoru Takahashi, Hiroko Yamashita, Yoshitaka Fujii

**Affiliations:** 1 Department of Oncology, Immunology and Surgery, Nagoya City University Graduate School of Medical Sciences, 1 Kawasumi, Mizuho-cho, Mizuho-ku, Nagoya 467-8601, Japan; 2 Department of Breast and Endocrine Surgery, Kumamoto University Graduate School of Medical Sciences, 1-1-1 Honjo, Kumamoto 860-8556, Japan; 3 Department of Breast Oncology, Aichi Cancer center Hospital, 1-1 Kanokoden, Chikusa-ku, Nagoya 464-8681, Japan; 4 Department of Experimental Pathology and Tumor Biology, Nagoya City University Graduate School of Medical Sciences, 1 Kawasumi, Mizuho-cho, Mizuho-ku, Nagoya 467-8601, Japan; 5 Division of Pathology, Nagoya City University Hospital, 1 Kawasumi, Mizuho-cho, Mizuho-ku, Nagoya 467-8601, Japan

## Abstract

**Introduction:**

Single-nucleotide polymorphisms (SNPs) in codon 72 of the *TP53 *(also known as *p53*) gene (rs1042522) and in the promoter region of the *MDM2 *gene (SNP309; rs2279744) have been suggested to play roles in many cancers. We investigated whether these SNPs were associated with patient outcome and the effect of adjuvant systemic therapy.

**Methods:**

The genotypes of *TP53 *codon 72 and *MDM2 *SNP309 were defined among 557 primary Japanese breast cancer patients (median follow-up, 61.7 months). The effects of several variables on survival were tested by Cox's proportional hazards regression analysis.

**Results:**

We showed that the Pro/Pro genotype of *TP53 *codon 72 was associated with poorer disease-free survival (DFS) than other genotypes by Kaplan-Meier analysis (*P *= 0.049) and multivariate Cox's proportional hazards regression analysis (*P *= 0.047, risk ratio of recurrence = 1.67), whereas *MDM2 *SNP309 status was not associated with DFS. The association of the Pro/Pro *TP53 *genotype with poorer DFS was especially significant in patients who received adjuvant chemotherapy (*P *= 0.009). In contrast, among the patients who had received adjuvant hormonal therapy or no adjuvant systemic therapy, *TP53 *codon 72 genotype was not associated with DFS.

**Conclusion:**

The Pro/Pro genotype of *TP53 *codon 72 appears to be an independent prognostic marker in breast cancer patients.

## Introduction

The *TP53 *tumor suppressor pathway is well-known to be crucial for maintaining genomic integrity and preventing cells from undergoing oncogenic transformation [1,2]. MDM2 plays a key role in regulating the *TP53 *pathway by binding directly to the p53 protein, inhibiting its activity and mediating degradation via the ubiquitination system [3]. p53 also positively regulates MDM2 expression, thereby creating a negative feedback loop [3]. Overexpression of MDM2 is observed both in epithelial cells of transgenic mice with induced mammary carcinomas [4] and in multiple human tumors, including breast cancer [5-7].

The *TP53 *codon 72 Arg>Pro (CGC to CCC) polymorphism of exon 4 [8] (National Center for Biotechnology Information single-nucleotide polymorphism (SNP) identification number rs1042522) has been suggested to play a role in several different cancer types. These two variant protein forms may behave differently, as the Arg/Arg genotype has been reported to induce apoptosis more effectively than the Pro/Pro genotype [9,10], which may be due to enhanced mitochondrial localization of p53 protein in cells with the Arg/Arg genotype [9]. In contrast, the Pro/Pro genotype appears to induce a higher level of G1 arrest than the Arg/Arg genotype [11,12]. Patients with the Pro/Pro genotype of *TP53 *in breast cancers have poorer survival than those with other genotypes [13]. Furthermore, retention of the Arg allele of *TP53 *in tumor tissue of Arg/Pro heterozygous breast cancer patients has been associated with reduced disease-free and overall survival [14]. Taiwanese lung cancer patients and Israeli colorectal cancer patients with the Pro/Pro genotype of *TP53 *also showed poorer survival [15,16]. A recent study showed that breast cancer patients with the Pro/Pro genotype were less sensitive to chemotherapy than those with Arg/Arg or Arg/Pro genotypes [17]. Similar results were reported in head and neck carcinoma [11]. On the other hand, estrogen receptor (ER) positive patients possessing the Pro allele had better distant recurrence-free survival when randomized to tamoxifen compared to those who did not receive tamoxifen, while homozygous Arg/Arg patients did not [18]. After the initial report of a statistically significantly increased risk of breast cancer in women homozygous for the Pro allele [19], numerous studies examined a possible role of this *TP53 *polymorphism in breast cancer risk. Meta-analysis of nine studies has recently shown that this *TP53 *polymorphism is not associated with breast cancer risk [20].

A SNP in the promoter of the *MDM2 *gene, referred to as SNP309 (a T→G change) (rs2279744), has been implicated in earlier age of onset of Li-Fraumeni syndrome and sporadic cancers [21]. The *MDM2 *SNP309 G/G homozygous genotype elevates MDM2 protein expression [21]. A recent study showed that cells that harbor this genotype had a compromised *TP53 *response pathway and formed transcriptionally inactive p53-MDM2 complexes in response to stress [22]. The G/G genotype was also associated with increased incidence of esophageal squamous cell carcinoma [23]. Colorectal cancer patients who had both the SNP309 G allele and wild-type *TP53 *were diagnosed at a younger age than those with the T/T genotype and wild-type *TP53 *[24]. On the other hand, no association was found between SNP309 status and breast cancer incidence [25-27]. However, a recent study showed that, in women whose breast cancers expressed high levels of ER, those having the *MDM2 *SNP309 G/G genotype showed earlier age of onset than those with the T/T genotype [28]. Another study showed that among patients with the T/T genotype, mutant status of *TP53 *and aberrant p53 protein expression were associated with poor survival, suggesting an interaction between *MDM2 *SNP309 and tumor *TP53 *status [25].

Here, we have examined germ-line DNA samples from 557 consecutive primary breast cancer patients to investigate the association between these SNPs and breast cancer prognosis.

## Materials and methods

### Study subjects

Blood samples were obtained from a total of 557 consecutive primary breast cancer patients at Nagoya City University Hospital, Nagoya, Japan, between January 1983 and December 2003. Informed consent was obtained from all patients before surgery. The histological grade was estimated according to the Bloom and Richardson method proposed by Elston and Ellis [29]. Treatment decisions were based on consensus recommendations at that time in Japan. After surgery, 77 patients received no adjuvant therapy. A total of 137 patients received adjuvant chemotherapy alone (64 received oral 5-fluorouracil (5-FU), 41 were treated with a CMF (cyclophosphamide/methotrexate/5-FU)-based regimen, 26 with an anthracycline-based regimen, and 6 received other regimens). A total of 195 patients received adjuvant hormonal therapy alone (157 received tamoxifen, 28 tamoxifen plus luteinizing hormone-releasing hormone (LH-RH) agonist, 5 were treated with LH-RH alone, and 5 with aromatase inhibitor). A total of 144 patients received a combination of hormonal therapy and chemotherapy. The adjuvant therapy received by four patients could not be determined due to missing records. Patients were followed postoperatively every three months for the first five years, then annually. The median follow-up period was 61.7 months (range 3 to 258 months). Relapse data were available in 497 of 557 patients examined: 105 (21.1%) experienced disease recurrence; 95 (19.1%) showed distant relapse; and 58 (11.6%) had died. This protocol was approved by the Institutional Review Board of Nagoya City University Graduate School of Medical Sciences.

### DNA extraction and genotyping

Genomic DNA was isolated from peripheral blood lymphocytes using a phenol-chloroform extraction method. Samples of genomic DNA were genotyped for *TP53 *codon 72 and *MDM2 *SNP309 polymorphisms. Genotyping was performed using TaqMan SNP genotyping assays. One *TP53 *probe (CTGCTCCCCGCGTGGCCC) was labeled at the 5'-end with VIC (Applied Biosystems' proprietary dye) and at the 3'-end with TAMRA. The other (CTGCTCCCCCCGTGGCCC) was labeled at the 5'-end with 6-carboxyfluorescein (FAM) and at the 3'-end with TAMRA. The *TP53 *primers were: forward, 5'-ATGAAGCTCCCAGAATGC-3', and reverse, 5'-GCCGGTGTAGGAGCT-3'. One *MDM2 *probe (CGCTGCGGCGCGGGA) was labeled at the 5'-end with VIC and labeled at the 3'-end with TAMRA. The other (CCGCTTCGGCGCGGGA) was labeled at the 5'-end with FAM and at the 3'-end with TAMRA. The *MDM2 *primers used for genotyping and sequencing were: forward, 5'-ATTTCGGACGGCTCTCGC-3', and reverse, 5'-GCGCAGCGTTCACACTAGTG-3'. Real-time TaqMan PCR and genotyping analyses were performed on an Applied Biosystems 7500 real-time PCR system (Applied Biosystems, Foster City, CA, USA) according to the manufacturer's standard PCR protocol. The results were analyzed on a 7500 real-time PCR system using the allelic discrimination assay program of Sequence Detection software version 1.3.1 (Applied Biosystems).

### DNA sequencing

DNA sequencing was performed to confirm the results of TaqMan SNP genotyping assays. The primers used for sequencing of *TP53 *were: forward, 5'-CCCGGACGATATTGAACAATGG-3', and reverse, 5'-CAGAATGCAAGAAGCCCAGAC-3'. As mentioned above, the primers used for sequencing of *MDM2 *were the same as those utilized in the TaqMan genotyping assays. The PCR products were sequenced by an ABI PRISM 3100 Genetic Analyzer and analyzed by ABI SeqScape Software version 2.1.1 (Applied Biosystems).

### Immunohistochemical analysis for p53

Serial sections (4 μm) for p53 staining were prepared from 289 consecutive breast cancer tissue blocks from 1983 to 1999 at Nagoya City University Hospital [30]. A monoclonal mouse antihuman p53 protein antibody (PAb1801; Novocastra, Newcastle, UK) was used at a 1:50 dilution. Bound antibody was detected with a streptavidin-biotin system (SAB-PO kit; Nichirei Co., Inc., Tokyo, Japan). Immunostaining was scored after the entire slide had been evaluated by light microscopy. The expression status of p53 was assessed according to the estimated proportion of tumor cells displaying positive nuclear staining. Scoring criteria for p53 protein expression were as follows (in the form, proportion of nuclear staining = score): none = 0, <1/10 = 1, 1/10 to 1/2 = 2, and >1/2 = 3. Tumors with a score of 1 or greater were considered to be positive for p53 protein accumulation.

### Estrogen receptor measurement

Frozen tissue specimens were analyzed for ER by an enzyme immunoassay using ER EIA 'Abbott' (Dinabot, Tokyo, Japan). The cut-off ER level was less than 13 fmol/mg protein.

### Statistical analyses

All molecular and immunohistochemical analyses were performed blinded to clinical data. Statistical calculations were done with StatView-J 5.0 software (SAS Institute Inc., Cary, NC, USA). The relationship between the genotype frequency of *TP53 *codon 72 or *MDM2 *SNP309 and clinicopathological factors were assessed by χ^2 ^and Fisher's exact probability tests. Disease-free survival (DFS) curves were generated by the Kaplan-Meier method and verified by the log-rank test. Cox's proportional hazards regression analysis was used for univariate and multivariate analyses of prognostic values. Differences were considered significant when a *P *value < 0.05 was obtained.

## Results

### Distributions of *TP53 *and *MDM2 *polymorphisms

Each of the *TP53 *codon 72 genotypes (Arg/Arg, Arg/Pro, or Pro/Pro) and *MDM2 *SNP309 genotypes (T/T, T/G, or G/G) was clearly discriminated using TaqMan SNP genotyping assays. The allelic discrimination data from these assays were confirmed by direct sequencing of representative PCR products. We did not find an association between *TP53 *codon 72 or *MDM2 *SNP309 genotypes and clinicopathological features in our series (Tables 1 and 2).

**Table 1:** Association between *TP53 *codon 72 status and clinicopathological characteristics

	Total, n (percent)	Arg/Arg, n (percent)	Arg/Pro, n (percent)	Arg/Arg +Arg/Pro, n (percent)	Pro/Pro, n (percent)	*P*^a^
Patients	557 (100)	63 (11)	281 (51)	344 (62)	213 (38)	
Age (median, year) (*n *= 557)		55	57	56	58	
Tumor size (*n *= 557)						0.16
≤ 2 cm	202 (36)	17 (27)	99 (35)	116 (33)	86 (40)	
2 cm to ≤ 5 cm	286 (51)	37 (59)	151 (54)	188 (55)	98 (46)	
>5 cm	57 (10)	6 (9)	28 (10)	34 (10)	23 (11)	
Unknown	12 (2)	3 (5)	3 (1)	6 (2)	6 (3)	
Node status (*n *= 557)						>0.99
Negative	321 (58)	34 (54)	167 (60)	201 (58)	120 (56)	
Positive	198 (35)	25 (40)	99 (35)	124 (36)	74 (35)	
Unknown	38 (7)	4 (6)	15 (5)	19 (6)	19 (9)	
Histology (*n *= 557)						0.98
IDC	486 (87)	54 (86)	246 (87)	300 (87)	186 (87)	
ILC	20 (4)	2 (3)	10 (4)	12 (4)	8 (4)	
Others	51 (9)	7 (11)	25 (9)	32 (9)	19 (9)	
ER status (*n *= 557)						0.13
Positive	344 (62)	33 (52)	171 (61)	204 (59)	140 (66)	
Negative	181 (32)	25 (40)	95 (34)	120 (35)	61 (29)	
Unknown	32 (6)	5 (8)	15 (5)	20 (6)	12 (5)	
Grade (*n *= 289)						0.27
1	71 (24)	3 (9)	48 (32)	51 (28)	20 (19)	
2	147 (51)	21 (62)	67 (45)	89 (48)	59 (56)	
3	54 (19)	8 (23)	28 (19)	36 (19)	18 (17)	
Unknown	17 (6)	2 (6)	7 (4)	9 (5)	8 (8)	
p53 IHC (*n *= 289)						>0.99
Positive	72 (25)	15 (44)	29 (19)	44 (24)	28 (27)	
Negative	196 (68)	19 (56)	108 (72)	127 (69)	69 (66)	
Unknown	21 (7)	0 (0)	13 (9)	13 (7)	8 (7)	

^a^Arg/Arg and Arg/Pro genotypes compared with Pro/Pro genotype. ER, estrogen receptor; IDC, invasive ductal carcinoma; IHC, immunohistochemistry; ILC, invasive lobular carcinoma.

**Table 2:** Association between *MDM2 *SNP309 status and clinicopathological characteristics

	Total, n (percent)	T/T, n (percent)	T/G, n (percent)	T/T + T/G, n (percent)	G/G, n (percent)	*P*^a^
Patients	557 (100)	111 (20)	263 (47)	374 (67)	183 (33)	
Age (median, year) (*n *= 557)		57	56	56	58	
Tumor size (*n *= 557)						0.49
≤ 2 cm	202 (36)	49 (44)	88 (33)	137 (37)	65 (36)	
2 cm to ≤ 5 cm	286 (51)	53 (48)	139 (53)	192 (51)	94 (51)	
>5 cm	57 (10)	6 (5)	28 (11)	34 (9)	23 (12)	
Unknown	12 (2)	3 (3)	8 (3)	11 (3)	1 (1)	
Node status (*n *= 557)						0.63
Negative	321 (58)	59 (53)	159 (60)	218 (58)	103 (56)	
Positive	198 (35)	42 (38)	88 (34)	130 (35)	68 (37)	
Unknown	38 (7)	10 (9)	16 (6)	26 (7)	12 (7)	
Histology (*n *= 557)						0.44
IDC	486 (87)	97 (87)	233 (89)	330 (88)	156 (85)	
ILC	20 (4)	3 (3)	8 (3)	11 (3)	9 (5)	
Others	51 (9)	11 (10)	22 (8)	33 (9)	18 (10)	
ER status (*n *= 557)						0.14
Positive	344 (62)	68 (61)	153 (58)	221 (59)	123 (67)	
Negative	181 (32)	33 (30)	95 (36)	128 (34)	53 (29)	
Unknown	32 (6)	10 (9)	15 (6)	25 (7)	7 (4)	
Grade (*n *= 289)						0.19
1	71 (24)	19 (31)	36 (26)	55 (27)	16 (18)	
2	147 (51)	29 (47)	66 (47)	95 (47)	52 (60)	
3	54 (19)	10 (16)	29 (21)	39 (19)	15 (17)	
Unknown	17 (6)	4 (6)	9 (6)	13 (6)	4 (5)	
p53 IHC (*n *= 289)						>0.99
Positive	72 (25)	19 (31)	31 (22)	50 (25)	22 (25)	
Negative	196 (68)	41 (66)	95 (68)	136 (67)	60 (69)	
Unknown	21 (7)	2 (3)	14 (10)	16 (8)	5 (6)	

^a^T/T + T/G genotypes versus G/G genotype. ER, estrogen receptor; IDC, invasive ductal carcinoma; IHC, immunohistochemistry; ILC, invasive lobular carcinoma.

### *TP53 *and *MDM2 *genotypes and breast cancer survival

**Figure 1:**
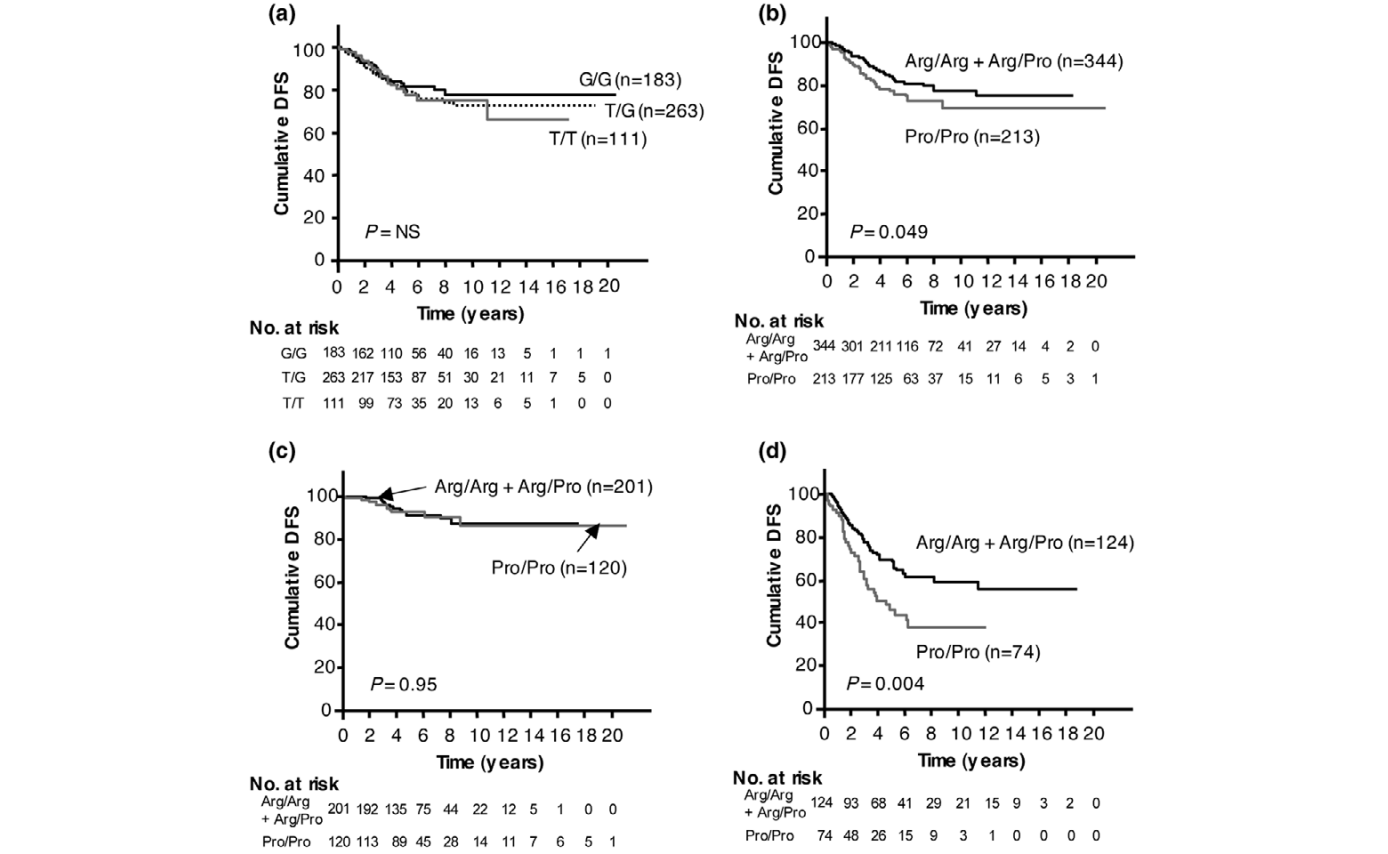
Kaplan-Meier survival curves of breast cancer patients. Disease-free survival (DFS) among patients categorized by **(a) ***MDM2 *SNP309 status and **(b) ***TP53 *codon 72 status. DFS in patients **(c) **without axillary lymph node involvement and **(d) **with lymph node metastasis according to their *TP53 *codon 72 status. NS, not significant.

We investigated whether these SNPs were associated with breast cancer survival. We found no association between *MDM2 *SNP309 status and DFS, as shown in Figure 1a. In contrast, the Pro/Pro genotype of *TP53 *codon 72 was significantly associated with poor DFS by Kaplan-Meier analysis, as shown in Figure 1b (*P *= 0.049), and multivariate Cox's proportional hazards regression analysis (*P *= 0.047, risk ratio of recurrence = 1.67, 95% confidence interval = 1.01 to 2.76) (Table 3). Our data indicates that the Pro/Pro genotype of *TP53 *codon 72 is an independent prognostic marker in breast cancer. In this analysis, we combined the Arg/Arg homozygous and the Arg/Pro heterozygous genotypes of *TP53 *codon 72, because the survival curve of the patients with the Arg/Arg genotype was similar to that of patients with the Arg/Pro genotype (data not shown). When the analysis was stratified by nodal status, no significant association between *TP53 *codon 72 genotype and DFS was seen for node-negative patients (*P *= 0.95; Figure 1c). In node-positive patients, however, the Pro/Pro genotype of *TP53 *codon 72 was significantly associated with poor DFS (*P *= 0.004; Figure 1d). Only 85 (26%) of 321 node-negative patients had received chemotherapy while 151 (76%) of 198 node-positive patients had. Therefore, we analyzed the relationship between breast cancer survival and this *TP53 *polymorphism in subgroups based on the type of adjuvant therapy.

**Table 3:** Uni- and mutivariate Cox's model (disease-free survival) of prognostic factors

		Univariate	Multivariate	
			
Variables	n	*P *value	*P *value	RR of recurrence (95 percent CI)
*TP53 *codon 72 status	557			
Arg/Arg or Arg/Pro				1.0 (referent)
Pro/Pro		0.049	0.047	1.67 (1.01 to 2.76)
*MDM2 *SNP309 status	557			
T/T or T/G				1.0 (referent)
G/G		0.41	0.99	1.00 (0.57 to 1.75)
Tumor size	545			
<2 cm				1.0 (referent)
≥2 cm		0.001	0.69	1.14 (0.61 to 2.12)
Lymph node	519			
Negative				1.0 (referent)
Positive		<0.0001	<0.0001	4.09 (2.35 to 7.12)
ER status	525			
Positive				1.0 (referent)
Negative		<0.0001	0.002	2.48 (1.40 to 4.39)
Grade	272			
1				1.0 (referent)
2 or 3		0.009	0.29	1.50 (0.71 to 3.14)
p53 IHC status	268			
Negative				1.0 (referent)
Positive		0.18	0.14	0.64 (0.35 to 1.16)

CI, confidence interval; ER, estrogen receptor; IHC, immunohistochemistry; RR, risk ratio.

### *TP53 *and *MDM2 *genotypes and adjuvant systemic therapy

**Figure 2:**
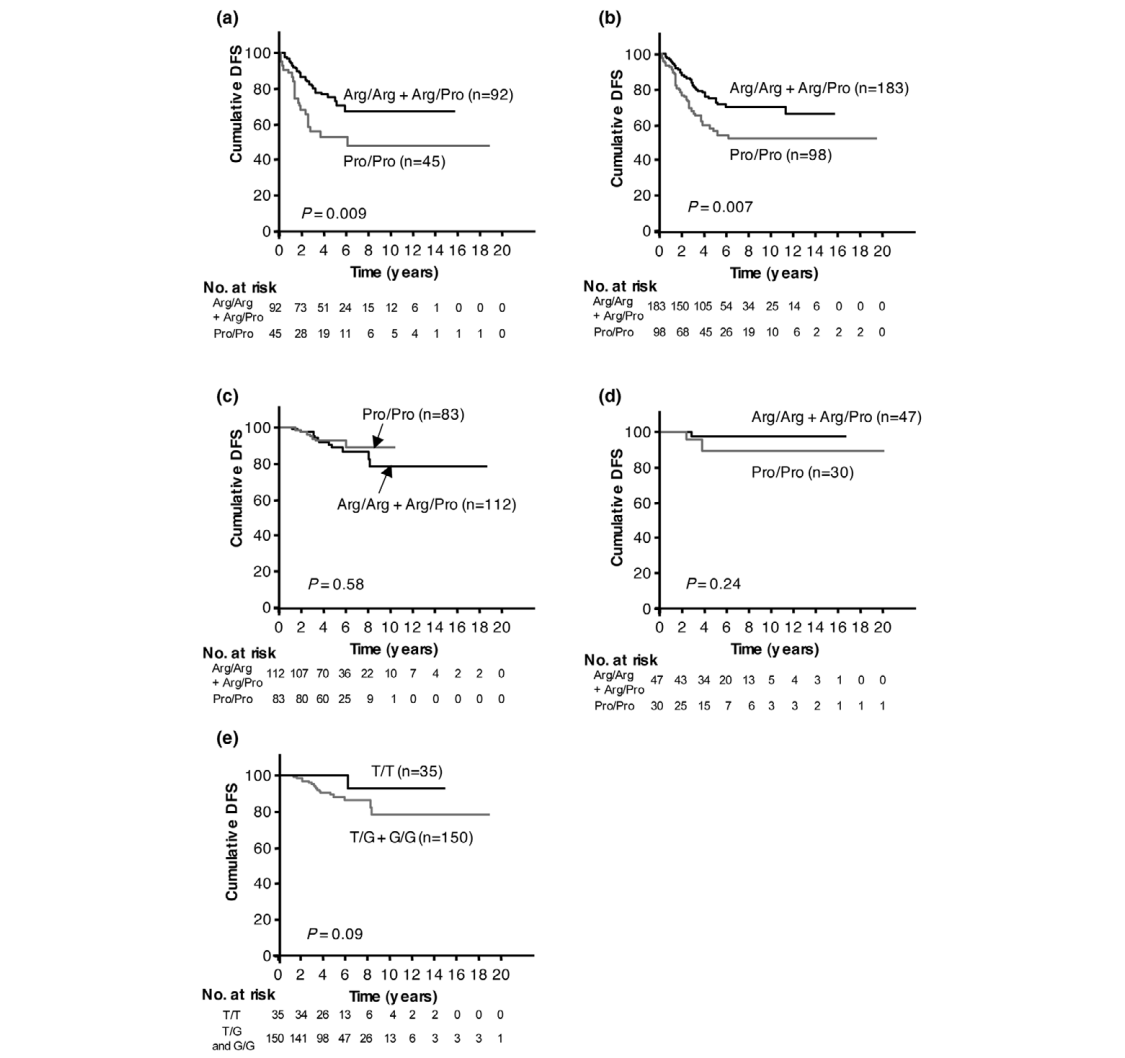
The effect of adjuvant systemic therapy on prognostic impact of polymorphisms. **(a) ***TP53 *codon 72 and adjuvant chemotherapy alone (*n *= 137); **(b) ***TP53 *codon 72 and adjuvant chemotherapy with or without hormonal therapy (*n *= 281); **(c) ***TP53 *codon 72 and adjuvant hormonal therapy alone (*n *= 195); **(d) ***TP53 *codon 72 and no adjuvant systemic therapy (*n *= 77); **(e) ***MDM2 *SNP309 and adjuvant tamoxifen with or without luteinizing hormone-releasing hormone analog (*n *= 185). DFS, disease-free survival.

Previous *in vitro *studies showed that cells with the *TP53 *codon 72 Arg/Arg genotype induced apoptosis markedly better than those with the Pro/Pro genotype [9,11]. A recent clinical study also suggested that breast cancer patients with the Pro/Pro genotype might be less sensitive to neoadjuvant chemotherapy than those with the Arg/Arg or Arg/Pro genotype [17]. Therefore, we hypothesized that this polymorphism might alter the sensitivity of tumors to adjuvant therapeutic agents. We analyzed the association between *TP53 *codon 72 polymorphism and DFS under different types of adjuvant systemic therapy. Our results show that, among patients who had received adjuvant chemotherapy alone (*n *= 137), those with the Pro/Pro genotype had poorer DFS than those with the Arg/Arg or Arg/Pro genotype, according to a Kaplan-Meier survival analysis (*P *= 0.009; Figure 2a). Similar results were observed when the analysis was expanded to include all patients receiving chemotherapy, with or without hormonal therapy (*n *= 281, *P *= 0.007; Figure 2b). In contrast, *TP53 *codon 72 genotype was not associated with DFS among patients who had received adjuvant hormonal therapy without chemotherapy (*n *= 195, *P *= 0.58; Figure 2c) nor among those who had received no adjuvant systemic therapy (*n *= 77, *P *= 0.24; Figure 2d).

A recent study proposed a model in which an estrogen-signaling pathway allows the G-allele of *MDM2 *SNP309 to accelerate breast cancer formation [28]. We hypothesized that this allele might also alter the sensitivity of tumors to adjuvant hormonal therapy. We therefore examined the correlation between *MDM2 *SNP309 and prognosis in patients who received adjuvant tamoxifen with or without LH-RH agonist. Although the T/T genotype tended to be associated with better DFS compared to other genotypes of SNP309 (Figure 2e), this association did not achieve significance (*P *= 0.09), and no statistically significant correlation was found between prognosis and *MDM2 *SNP309 genotype.

## Discussion

We demonstrate here that SNPs of *TP53 *codon 72 may have an important role in breast cancer. We show that the Pro/Pro genotype at codon 72 of *TP53 *was associated with poor DFS, particularly in patients who received adjuvant chemotherapy while *MDM2 *SNP309 was not associated with prognosis.

*TP53 *codon 72 encodes two distinct functional allelic forms: arginine (Arg) or proline (Pro) [8]. Polymorphism at this codon has been suggested to modulate *TP53*-dependent apoptosis and modify sensitivity to chemotherapeutic agents [9,11,12,17]. Recent studies reported that the Pro/Pro genotype of *TP53 *codon 72 was associated with poorer survival in Finnish breast cancer patients [13], and suggested that *MDM2 *SNP309 status is associated with p53 protein function [21,22]. These findings inspired us to investigate the association of breast cancer prognosis with SNPs of *TP53 *codon 72 and *MDM2 *SNP309. We used the TaqMan SNP genotyping assay, which is amenable to high-throughput genotyping and avoids many problems of traditional genotyping assays, such as PCR-restriction fragment length polymorphism [31]. Although the TaqMan assay is convenient and reliable, it is less accurate than direct sequencing. Therefore, we analyzed our data carefully. Our results show that the Pro/Pro genotype of *TP53 *was associated with poorer DFS in Japanese breast cancers patients, thus supporting the Finnish study mentioned above [13]. However, the authors of that study did not address the question of why the Pro/Pro genotype of *TP53 *adversely affected the prognosis of breast cancer patients. Analysis of our entire set of 557 patients showed a relationship between the *TP53 *Pro/Pro genotype and DFS, but with a *P *value (0.047) at the borderline of significance, leaving doubt as to whether the Pro/Pro genotype is an independent risk factor for poor DFS.

We therefore attempted to identify subgroups in which the effect of the *TP53 *codon 72 was more significant. A recent study showed that breast cancer patients with the Pro/Pro genotype demonstrated less sensitivity to a neoadjuvant chemotherapy regimen that included 5-FU, cyclophosphamide, and the anthracycline derivative pirarubicin [17]. This suggested that *TP53 *codon 72 polymorphism might be a strong predictive marker for chemotherapy response in breast cancer patients. Our data show that the Pro/Pro genotype was associated with poor DFS in node-positive but not in node-negative patients, and 76% of node-positive patients in our series had received adjuvant chemotherapy while only 26% of node-negative patients had.

Although lymph node status is an important factor in classical staging of breast cancer based on histological/anatomical markers, there is little evidence that breast cancers with lymph node involvement are biologically different from those without it [32-34]. We therefore considered that the apparent effect of node status on the relationship between *TP53 *genotype and node status was due to a correlation between codon 72 polymorphism and effect of adjuvant chemotherapy, since 76% of node-positive patients in our series had received such therapy while only 26% of node-negative patients had. We thus analyzed breast cancer survival with respect to this *TP53 *polymorphism and the type of adjuvant therapy administered. Our data show that among patients who had received adjuvant chemotherapy, those with the Pro/Pro genotype of *TP53 *exhibited poorer DFS. Our finding is also consistent with a previous study of head and neck carcinoma showing that among patients who had received chemo-radiotherapy, those with the Pro/Pro genotype of *TP53 *showed poorer survival compared to patients with other genotypes [11].

Moreover, in ovarian cancer patients who received adjuvant cisplatinum/paclitaxel chemotherapy, the *TP53 *Pro allele was associated with a poorer prognosis [35]. An *in vitro *study showed that anticancer agents such as doxorubicin, 5-FU, and cisplatin induced a higher level of apoptosis in human H1299 cells expressing the Arg/Arg genotype of *TP53 *codon 72 than in those expressing the Pro/Pro genotype [11]. In addition, in a colony-survival assay, doxorubicin and cisplatin were more cytotoxic to cells expressing the Arg variant than to those expressing the Pro variant [11]. Our data are consistent with this *in vitro *study. The results among patients receiving both chemotherapy and hormonal therapy were similar to those among patients who had received adjuvant chemotherapy alone (data not shown), but no correlation was found between *TP53 *polymorphism and DFS among the patients receiving adjuvant hormonal therapy alone. Positive correlation between *TP53 *polymorphism and DFS was observed among patients receiving any chemotherapeutic agents. Since these compounds are cytotoxic while hormonal therapeutic agents are cytostatic, our results may reflect differential effects of *TP53 *polymorphism on different apoptotic pathways. Recently, ER positive patients possessing the Pro allele of *TP53 *codon 72 have been reported to show better distant recurrence-free survival when randomized to tamoxifen compared to those not receiving tamoxifen [18]. That report suggested that the Pro allele of *TP53 *codon 72 might be a predictor of tamoxifen response [18], although the authors did not present a Kaplan-Meier analysis of DFS by *TP53 *codon 72 genotype among patients receiving tamoxifen. We did not find any correlation between the genotype of *TP53 *codon 72 and prognosis in patients receiving tamoxifen alone (data not shown).

Neither did we find any statistically significant association between *MDM2 *SNP309 and survival in any subgroups or in the total population, although we did observe a non-significant tendency toward better DFS with the T/T genotype in patients who received adjuvant tamoxifen with or without LH-RH analog. Bond *et al. *[28] proposed a model in which an estrogen-signaling pathway allows the G-allele of *MDM2 *SNP309 to accelerate breast cancer formation. This allele might also alter the efficacy of tamoxifen, although the mechanism is unclear. A recent report showed that the G/G genotype of *MDM2 *SNP309 was associated with poor prognosis, as well as *TP53 *mutations and p53 protein immunopositivity, in gastric carcinoma [36]. *TP53 *alteration is correlated with shortened survival of patients with gastric carcinoma [37,38]. This suggests that the G/G genotype of *MDM2 *SNP309 might, therefore, be predictive of poor survival in gastric carcinoma patients. Many reports demonstrate that *TP53 *mutations confer a worse overall and disease-free survival in breast cancer patients [39,40], while the prognostic value of p53 protein accumulation has not been consistently demonstrated [41,42]. Although *TP53 *mutation status was not available in our series, we analyzed the correlation between *MDM2 *SNP309 and p53-immunopositivity, but did not find any association between them.

In previous reports, the frequency of the Pro/Pro genotype of *TP53 *codon 72 was about 7% to 30% [13,15,17,18,23,43,44], although 39.1% of patients with signet ring cell gastric carcinomas and 37.7% of female patients with lung carcinomas had the Pro/Pro genotype in subgroup analyses [15,44]. The reported frequency of the G/G genotype of *MDM2 *SNP309 is about 10% to 30% [23-28,36,43]. We found that 38% of patients had the Pro/Pro genotype of *TP53 *codon 72 and 33% had the G/G genotype of *MDM2 *SNP309. Therefore, the allelic discrimination data from TaqMan SNP genotyping assays were confirmed by direct sequencing of representative PCR products.

One of the goals of translational cancer research is to identify molecular predictors of chemotherapy treatment. Molecular genetic determinants of treatment outcome are important to facilitate identification of patients most likely to benefit from chemotherapy. The present results suggest that the SNP of *TP53 *codon 72 is a potentially useful marker. However, this is a retrospective pilot study. Further work will be needed to verify the effect of this SNP in breast cancer.

## Conclusion

The genotypes of *TP53 *codon 72 and *MDM2 *SNP309 were defined among 557 primary Japanese breast cancer patients. Our data show that the Pro/Pro genotype of *TP53 *codon 72 appears to be an independent prognostic marker in breast cancer patients.

## Abbreviations

DFS = disease-free survival; ER = estrogen receptor; FU = fluorouracil; LH-RH = luteinizing hormone-releasing hormone; SNP = single-nucleotide polymorphism.

## Competing interests

The authors declare that they have no competing interests.

## Authors' contributions

TT conceived the design, carried out genotyping assays and drafted the manuscript. ZZ carried out genotyping assays and DNA sequencing. MN carried out genotyping assays. MH performed the statistical analysis. NK collected the patients' data. H Iwase and ST provided study material. H Iwata participated in the study design. HY participated in the study design. YF supported the study financially. All authors read and approved the final manuscript.
